# Comparison of an Automated System with Conventional Identification and Antimicrobial Susceptibility Testing

**DOI:** 10.5402/2012/107203

**Published:** 2012-09-16

**Authors:** Shalini Duggal, Rajni Gaind, Neha Tandon, Manorama Deb, Tulsi Das Chugh

**Affiliations:** ^1^Department of Microbiology, Dr. Baba Saheb Ambedkar Hospital, Rohini, New Delhi 110085, India; ^2^Department of Microbiology, VMMC and Safdarjung Hospital, New Delhi 110029, India; ^3^Department of Microbiology, BLK Super Speciality Hospital, New Delhi 110005, India

## Abstract

The present study was designed to compare a fully automated identification/antibiotic susceptibility testing (AST) system BD Phoenix (BD) for its efficacy in rapid and accurate identification and AST with conventional manual methods and to determine if the errors reported in AST, such as the (very major errors) VME (false susceptibility), (major errors) ME (false resistance), and (minor errors) MiE (intermediate category interpretation) were within the range certified by FDA. Identification and antimicrobial susceptibility test results of eighty-five clinical isolates including both gram-positive and negative were compared on Phoenix considering the results obtained from conventional manual methods of identification and disc diffusion testing of antibiotics as standards for comparison. Phoenix performed favorably well. There was 100% concordance in identification for gram-negative isolates and 94.83% for gram-positive isolates. In seven cases, Phoenix proved better than conventional identification. For antibiotic results, categorical agreement was 98.02% for gram-positive and 95.7% for gram-negative isolates. VME was 0.33%, ME 0.66%, MiE 0.99% for gram-positive isolates and 1.23% VME, 1.23% ME, and 1.85% MiE for gram-negative isolates. Therefore, this automated system can be used as a tool to facilitate early identification and susceptibility pattern of aerobic bacteria in routine microbiology laboratories.

## 1. Introduction

Two million people in India die each year due to infectious diseases [1]. There is a need to integrate medicine and innovative technology in our public health system to provide rapid, efficient, accurate, and cost-effective results for identification and antimicrobial susceptibility testing (AST) of pathogens. Automated identification/AST systems can aid in rapid diagnosis of bacterial pathogens. Since Phoenix (BD) was first installed in India at our institute, a comparison study to evaluate it in reference to conventional manual methods was done. 

## 2. Material and Methods

Eighty-five clinical isolates were studied on Phoenix (BD Diagnostics, Gurgaon, India) of which 58 were gram-positive (PMIC panels) and 27 were gram-negative isolates (NMIC panels). Isolates were processed directly from the primary plates, and purity testing was done simultaneously by standard methods. Identification by conventional methods was confirmed on the basis of results obtained by performing routine biochemical tests [2] and AST was performed by the disc diffusion test based on Kirby-Bauer method in compliance with CLSI guidelines [3]. Identification in Phoenix was based on colorimetric and fluorometric reactions while the AST was based on turbidimetry and redox reactions [4]. Manually, nine biochemical tests could be done for identification of gram-negative bacilli and three for gram-positive cocci. Phoenix panels include 45 biochemicals and 20 antibiotics with MIC (minimum inhibitory concentration) for gram-negative and 46 biochemicals and 22 antibiotics with MIC for gram-positive isolates. The conventional manual tests were catalase, oxidase test, indole test, urease production, citrate utilization, glucose and lactose fermentation, triple sugar iron media, and lead acetate for hydrogen sulfide production in gram-negative isolates and catalase, coagulase, and bile esculin production for gram-positive isolates. Reagents for these biochemical tests were supplied by Himedia Laboratories, Mumbai, India. Discordant results between Phoenix and conventional methods were recorded and compared. Reproducibility of testing in Phoenix was tested in eighteen randomly selected isolates. For data analysis, 5 antibiotics including ampicillin, levofloxacin, vancomycin, linezolid, and nitrofurantoin were compared in enterococci and erythromycin, clindamycin, nitrofurantoin, moxifloxacin, vancomycin, linezolid, trimethoprim/sulfamethoxazole, and oxacillin in staphylococci. For gram-negative isolates, amikacin, amoxicillin/clavulanic acid, ceftazidime, ciprofloxacin, imipenem, piperacillin/tazobactam, cefepime, ceftriaxone, cefuroxime, trimethoprim/sulfamethoxazole, aztreonam and nitrofurantoin were compared. The antibiotic discs were supplied by BD BBL Sensi-disc, Gurgaon, India. Categorical agreement [5] which is defined as susceptible, intermediate, and resistant results after matching two different systems was determined considering disc diffusion results as standard. *Staphylococcus aureus *ATCC 25923, *Enterococcus faecalis *ATCC 29212, *Escherichia coli* ATCC 25922, *Pseudomonas aeruginosa *ATCC 27853, and ESBL producing *Klebsiella pneumoniae *ATCC 700603 were used as quality control (QC) standard strains (BD, Gurgaon, India) for both methods. The antibiotic discs were tested once weekly with these strains and the Phoenix panels were tested with respective ATCC strains after any new lot was received.

## 3. Results

Both methods were comparable with ATCC control strains and the QC tests showed correct results. The results were compared by entering the data on excel sheets and simple statistical calculations were made and recorded. 

Fifty-eight gram-positive isolates were tested including six staphylococci, 51 enterococci, and one *Streptococcus* group B. Three isolates including two isolates of coagulase-negative *Staphylococcus* (CoNS) and one *Enterococcus *spp. showed discordant identification between Phoenix and conventional methods. Phoenix could identify seven isolates more accurately to species level. These isolates included strains of *Enterococcus durans, Staphylococcus capitis *ssp* capitis, *and* Leuconostoc lactis* which were manually identified as *Enterococcus *spp. but correlated with Phoenix reports after testing with more biochemicals. AST reported by Phoenix was mostly in agreement with the disc diffusion test except for one isolate of *S. saprophyticus* and 3 isolates of enterococci which gave discrepant results for one or more antimicrobials (Table 1). Streptococcal susceptibility was not in Phoenix database for the PMIC panel used, therefore, could not be compared. Among 303 antimicrobial results compared, categorical agreement was seen between Phoenix and the disc diffusion method in all except 6 combinations (98.02%). Notably, there was one VME (0.33%), 2 ME (0.66%), and 3 MiE (0.99%).

Twenty-seven gram-negative isolates were tested including members of family Enterobacteriaceae, *Acinetobacter* spp., *Pseudomonas *spp. and other nonfermenters. Concordance in identification was 100% up to genus level, for one isolate, species was correctly identified by Phoenix but not by manual biochemicals. Fourteen discordant results were obtained out of 324 results obtained by comparing results for 12 antimicrobials in 27 isolates. Therefore, categorical agreement between Phoenix and disc diffusion method was 95.7% which included 4 VME (1.23%), 4 (1.23%) ME, and 6 MiE (1.85%) (Table 1).

Average time to final identification and susceptibility result for all the isolates was 11 hr. Phoenix gave alert values like MRS (methicillin-resistant *Staphylococcus*), VRE (vancomycin-resistant *Enterococcus*), ESBL (extended spectrum beta-lactamase) producer, HLAR (high-level aminoglycoside-resistance) and for intrinsic resistance in isolates specific group of drugs.

To check the consistency and reproducibility of Phoenix results, 18 random strains were tested twice in Phoenix. The results are depicted in Table 2. There were a total of two identification errors (11.1%) and six errors in categorical agreement among 105 isolate-antibiotic combinations tested (5.7%).

## 4. Discussion

Phoenix gave rapid results with alert values like MRS, VRE, and ESBL if detected. The average time to result was 11 hours for identification and AST which is much less compared to the conventional methods and hence reduced turnover time with increased accuracy. When opted for “critical test”, results could be even faster as early as 6 hr. In our study, the overall agreement for identification by Phoenix as compared to manual method was 94.83% with 100% agreement for gram-negative bacteria. Also, Phoenix was able to identify the isolates to species level with >95% confidence. Phoenix was also found to be more accurate in identifying seven gram-positive isolates and one gram-negative isolate compared to the manual method. 

For AST, the errors were within the range specified by FDA that major error rate must be less than 3% of all the susceptible organisms tested and very major error rate 1.5% or less [5]. Also, the categorical agreement (CE) should be ≥90% when two systems are compared [5, 6] which was very well seen with gram-positive and -negative isolates. CE should be ≥95% on reproducibility testing [6], in our study it was slightly less but that could have been because of less number of isolates chosen for testing reproducibility. In India, this is the first study comparing Phoenix system to conventional methods of identification and antibiotic susceptibility testing. Among studies done elsewhere, discrepancies in AST were mostly among beta-lactam group of drugs as observed in automated systems including Phoenix, Microscan walkaway, Vitek and Vitek 2 by Sader et al. [7] and Juretschko et al. [8]. Compared to other automated systems, Phoenix has been found to correlate well as reported by Brisse et al. [9], Dallas et al. [10] and Mittman et al. [11]. Compared to conventional manual methods, our results are comparable to two studies by Carroll et al. [12, 13], where categorical agreement among both gram-positive and -negative isolates was ≥97% each. However, local antibiogram must be kept in mind while reporting the results and doubtful cases must be repeated. 

Drawbacks in our study are that comparison of antimicrobial susceptibility has been done by MIC in Phoenix with zone diameters of disc diffusion method, and concordance of isolates is compared only up to genus level in some instances. Also, not all antibiotic sensitivities could be compared between the two methods.

To conclude, the Phoenix system is efficient for rapid identification and antimicrobial susceptibility testing of bacteria. The capability to rapidly identify and report resistance markers like ESBL, VRE, MRSA, which are critical, helps in adequate therapy and patient care. By providing faster results, it decreases antibiotic consumption and improves patient care.

## Figures and Tables

**Table 1:** Discordant results obtained for isolates by Phoenix as compared to Kirby-Bauer disk diffusion test.

Antimicrobial (*n*)*	Organism	Phoenix AST	Disc diffusion	Interpretation
Erythromycin (1)	*S. saprophyticus*	Susceptible	Resistant	Very major error
Vancomycin (1)	*Enterococcus* spp.	Resistant	Susceptible	Major error
Linezolid (2)		Intermediate	Susceptible	Minor errors
Erythromycin (1)		Intermediate	Susceptible	Minor error
Nitrofurantoin (1)		Resistant	Susceptible	Major error
Piperacillin/tazobactam (3)	*Escherichia coli*	Sensitive	Resistant	Very major error
Cefepime (2)	*Escherichia coli*	Sensitive	Resistant	Very major error
		Resistant	Intermediate	Minor error
Nitrofurantoin (1)	*Escherichia coli*	Intermediate	Susceptible	Minor error
Amikacin (3)	*Escherichia coli*	Resistant	Susceptible	Major error
		Susceptible	Intermediate	Minor error
	*Proteus mirabilis*	Resistant	Susceptible	Major error
Ciprofloxacin (2)	*Enterobacter cloacae*	Resistant	Intermediate	Minor error
	*Proteus mirabilis*	Resistant	Susceptible	Major error
Ceftriaxone (1)	*Morganella morganii*	Resistant	Intermediate	Minor error
Imipenem (1)	*Acinetobacter lowffii*	Resistant	Intermediate	Minor error
Ceftazidime (1)	*Proteus mirabilis*	Resistant	Susceptible	Major error

*(*n*): Total number of discordant results.

**Table 2:** Showing reproducibility of isolates on repeat testing in Phoenix.

Isolate	Difference in identification	Difference in AST	Type of error
*Staphylococcus aureus* ATCC 25923	None	None	—
*Pseudomonas aeruginosa* ATCC 27853	None	None	—
*S. saprophyticus*	*S. aureus*	None	Identification
*S. saprophyticus*	None	Chloramphenicol	Major error
*S. haemolyticus*	None	None	—
*Enterococcus faecalis*	None	Vancomycin	Major error
*Enterococcus faecium* (2)	None	Nitrofurantoin (2)	Minor error
		Moxifloxacin	Major error
*Enterococcus durans*	None	Gatifloxacin	Minor error
*Leuconostoc lactis*	None	None	—
*Streptococcus* group “B”	None	AST not in Phoenix database	—
*Streptococcus pneumoniae*	None	AST not in Phoenix database	—
*Streptococcus sanguinis*	None	AST not in Phoenix database	—
*Enterobacter cloacae*	None	Piperacillin/Tazobactam	Minor error
*Acinetobacter baumannii*	None	None	—
*Chromobacterium violaceum*	*Burkholderia cepacia*	AST not in Phoenix database	Identification
*Pseudomonas oryzihabitans*	None	AST not in Phoenix database	—
*Stenotrophomonas maltophilia*	None	None	—

None: no difference found.
